# Rapid and Sensitive Detection of Tomato Brown Rugose Fruit Virus in Tomato and Pepper Seeds by Reverse Transcription Loop-Mediated Isothermal Amplification Assays (Real Time and Visual) and Comparison With RT-PCR End-Point and RT-qPCR Methods

**DOI:** 10.3389/fmicb.2021.640932

**Published:** 2021-04-21

**Authors:** Domenico Rizzo, Daniele Da Lio, Alessandra Panattoni, Chiara Salemi, Giovanni Cappellini, Linda Bartolini, Giuseppe Parrella

**Affiliations:** ^1^Laboratory of Phytopathological Diagnostics and Molecular Biology, Plant Protection Service of Tuscany, Pistoia, Italy; ^2^Department of Agricultural, Food and Agro-Environmental Sciences, University of Pisa, Pisa, Italy; ^3^Institute for Sustainable Plant Protection of National Research Council (IPSP-CNR), Portici, Italy

**Keywords:** virus diagnostic, seeds contamination, seed testing, tomato brown rugose fruit virus, RT-LAMP assay

## Abstract

Tomato brown rugose fruit virus (ToBRFV) represents an emerging viral threat to the productivity of tomato and pepper protected cultivation worldwide. This virus has got the status of quarantine organism in the European Union (EU) countries. In particular, tomato and pepper seeds will need to be free of ToBRFV before entering the EU and before coming on the market. Thus, lab tests are needed. Here, we develop and validate a one-step reverse transcription LAMP platform for the detection of ToBRFV in tomato and pepper leaves, by real-time assay [reverse transcription loop-mediated isothermal amplification (RT-LAMP)] and visual screening (visual RT-LAMP). Moreover, these methods can also be applied successfully for ToBRFV detection in tomato and pepper seeds. The diagnostic specificity and sensitivity of both RT-LAMP and visual RT-LAMP are both 100%, with a detection limit of nearly 2.25 fg/μl, showing the same sensitivity as RT-qPCR Sybr Green, but 100 times more sensitive than end-point RT-PCR diagnostic methods. In artificially contaminated seeds, the proposed LAMP assays detected ToBRFV in 100% of contaminated seed lots, for up to 0.025–0.033% contamination rates in tomato and pepper, respectively. Our results demonstrate that the proposed LAMP assays are simple, inexpensive, and sensitive enough for the detection of ToBRFV, especially in seed health testing. Hence, these methods have great potential application in the routine detection of ToBRFV, both in seeds and plants, reducing the risk of epidemics.

## Introduction

Tomato brown rugose fruit virus (ToBRFV), a member of the genus *Tobamovirus*, family *Virgaviridae*, is an emerging and highly virulent virus, mainly affecting tomato crops worldwide. ToBRFV presents a genome organization common to tobamoviruses, with a single-strand positive-sense RNA of ~6,400 nucleotides (nt) with four open reading frames (ORFs) that encode two replication-related proteins (Salem et al., 2016). Since the first report of ToBRFV outbreak in tomato in Jordan (Salem et al., 2016), the virus has been identified within recent years in other countries on different continents: Israel (Luria et al., 2017), Mexico (Cambrón-Crisantos et al., 2018), United States (Chitambar, 2018; Ling et al., 2019), Germany (Menzel et al., 2019), Italy (Panno et al., 2019), Palestine (Alkowni et al., 2019), Turkey (Fidan, 2020), China (Yan et al., 2019), the United Kingdom (Skelton et al., 2019) and, more recently, in Greece (Beris et al., 2020). Likely occurrences have also been reported (but not confirmed) in Chile, Ethiopia, Sudan, and Netherlands.

Tomato brown rugose fruit virus constitutes an emerging threat of global concern to tomato crops, as it is able to overcome the resistance gene *Tm-2^2^* routinely used by breeders for the constitution of tomato hybrids, with special reference to those destined for protected cultivation, as it is effective in controlling other tomato tobamoviruses, such as tobacco mosaic virus (TMV) and tomato mosaic virus (ToMV; Zhang et al., 2013). On the other hand, pepper varieties harboring the *L1*, *L3*, or the *L4* alleles of the *L* resistance gene to tobamoviruses have displayed hypersensitivity response (HR) when inoculated with ToBRFV, allowing for some control of the virus. More recently, a severe outbreak of ToBRFV in a red sweet pepper (*Capsicum annum*) variety not harboring the resistance gene, has been recorded in Sicily (south Italy), with an incidence of the viral disease of about 85% (Panno et al., 2020b).

Seeds provide an efficient means for disseminating many plant diseases across the world. In particular, it has been widely documented that seed-transmitted viruses are often introduced into new countries and continents through infected germplasm, due to the global trade involving large-scale movements of seeds. Tobamoviruses are seed-borne, mechanically transmitted stable viruses (Dombrovsky and Elisheva, 2017). By analogy with other tobamoviruses infecting tomato and pepper (i.e., TMV and ToMV), the seed transmission of ToBRFV is strongly suspected but has not yet been definitely demonstrated (Dombrovsky and Elisheva, 2017). Nevertheless, the long-distance movement of ToBRFV by means of infected seeds could explain how this virus emerged so rapidly and simultaneously in different countries. Moreover, it is important to underline that, even if most of the tobamoviruses display a low percentage of seed transmission (primary infection), such very low occurrence of seed transmission is enough to cause an outbreak of the disease, as these viruses are easily transmitted mechanically through wounding, which is often caused by human activity during agronomic crop management, by contact with infected plants or facilitated by pollinator activity (secondary infection), as demonstrated recently just for ToBRFV (Levitzky et al., 2019; Panno et al., 2020a).

Given its rapid spread and potential harm to tomatoes and peppers, ToBRFV has been included in EPPO’s *Alert list* and has been regulated in the European Union since November 2019 (Commission Implementing Decision EU 2019/1615). In addition, ToBRFV has also been included in the list of quarantine bodies (Commission regulation – EU – 2019/2072), as well as included in the priorities of the European Union (Commission regulation – EU – 2019/1702). In light of European legislation, the cultivation of host plants must be subjected to territorial monitoring every year, in order to check for the presence of the virus. In particular, for tomato and bell pepper, specific measures have been approved, with the introduction and movement of the virus in the EU being prohibited. In particular, considering that the virus can be spread through the movement of seeds, tomato, and pepper seeds must be free of ToBRFV or originate from ToBRFV-free areas, both before entering the EU and before coming onto the market. Current ToBRFV outbreaks in different parts of the world highlight that early detection is crucial to prevent the spread of the virus, to control outbreaks and, eventually, to quickly begin appropriate eradication interventions. Therefore, rapid and effective detection methods are needed, particularly at entry points or during monitoring investigations, to prevent outbreaks in new environments with negative ecological and economic consequences. Phytosanitary certification systems have been established all over the world, in order to certify the propagation of virus-free plant material (EPPO, 2009). Implementing these disease control schemes requires techniques with high sensitivity and specificity, such as biomolecular methods.

Different techniques have been proposed to detect ToBRFV in seeds, leaves, or petioles, including biological indexing, serology, and nucleic acid-based detection techniques with specific primers such as reverse transcription polymerase chain reaction (RT-PCR), real-time RT-PCR, and *reverse transcription* loop-mediated isothermal amplification (RT-LAMP; Dombrovsky and Elisheva, 2017; Almeida et al., 2018; Panno et al., 2019). Although real-time RT-PCR assay presents increased sensitivity and stability, compared to conventional RT-PCR, this method still requires sophisticated and expensive equipment and reagents, which may not be available in laboratories with limited resources. This status has prompted motivation for the investigation and development of a sensitive but cost-effective alternative technique. Loop-mediated isothermal amplification (LAMP) is a sensitive and rapid nucleic acid amplification technology, first reported by Notomi et al. (2000). To date, LAMP has reached many fields of application, including plant pathology (Panno et al., 2020c). In particular, RT-LAMP is a valid substitute for RT-PCR, due to its simplicity, rapidity, specificity, and sensitivity, as only a water bath or thermoblock capable of ensuring a constant temperature (60–65°C) is required. The LAMP reaction is an auto-cycling strand amplification reaction based on the *Bacillus stearothermophilus* (*Bst*) DNA polymerase, which possesses strand-displacement activity, and two or three pairs of specific primers that recognize four or six stem-loop DNA regions with various lengths. The product of the LAMP reaction can be detected using agarose gel or an intercalator that emits fluorescence in the case of amplification in RT-LAMP. The latter can be visualized by monitoring either the turbidity using a photometer, the fluorescence using a fluorimeter, or by the naked eye under a UV lamp when using an intercalating dye which changes color (Panno et al., 2020c).

In this study, we have developed a rapid and sensitive RT-LAMP and a visual RT-LAMP assay for the specific detection of ToBRFV RNA for the first time in tomato and pepper seeds using a single-tube one-step RT-LAMP and visual RT-LAMP, as well as comparing the sensitivity and specificity of the developed methods with those of RT-PCR and real-time RT-PCR.

## Materials and Methods

### Virus Isolates and Plant Material

Four biologically and molecularly characterized ToBRFV isolates were used to evaluate the RT-LAMP assay. Isolates were obtained as lyophilized infected tomato leaves from various sources, both institutional and commercial: Isolate Sic1/19, from the University of Palermo (Italy); isolate T1101, from the Institute of Sustainable Plant Protection of the National Research Council (IPSP-CNR), Torino (Italy); isolate TBRFV-Ps1, from the An-Najah National University (Palestine); and isolate PC-1236, from North Rhine-Westphalia, Germany (DSMZ – German Collection of Micro-organisms and Cell Cultures).

All isolates were maintained, in an insect-proof greenhouse, on the tomato line Momor carrying the *Tm-2^2^* gene of resistance to tobamoviruses (Dombrovsky and Elisheva, 2017), and on the pepper ecotype Friariello, without any resistance gene. Healthy tomato and pepper plants were also maintained in a separate greenhouse compartment and used as controls. To check the specificity of the RT-LAMP assay, additional tobamoviruses, obtained from the DSMZ collection as dehydrated leaves, were included in the trials for method validation. In particular, bell pepper mottle virus (BPeMV, isolate PC-0170), odontoglossum ringspot virus (ORSV, isolate PC 0625), paprika mild mottle virus (PaMMV, isolate PC 0606), pepper mild mottle virus (PMMoV, isolate PC 0165), streptocarpus flower break virus (SFBV, isolate PC 1058), tobacco mild green mosaic virus (TMGMV, isolate PC 0887), ToMV (isolate PC 15705), TMV (isolate PC 0107), turnip vein clearing virus (TVCV, isolate PC 0148), youcai mosaic virus (YMoV, isolate PC 0527), and watermelon chlorotic stunt virus (WmCSV, isolate PC 0830) were used.

Finally, several batches of uncontaminated tomato and pepper seeds were used for specific ToBRFV detection tests in tomato and pepper seeds (see below).

### LAMP Primer Design

**Figure 1:**
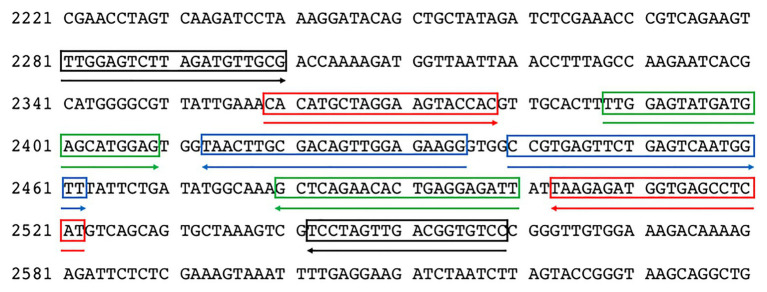
Distribution of the LAMP primers on the nucleotide sequence of ToBRFV (GenBank Acc. Number MN815773). F3/B3 primers are highlighted in black, F2/B2 in red, LoopF/LoopB in green, and F1c/B1c in blue.

Nine complete ToBRFV genome sequences available in the NCBI GenBank^1^ were downloaded and aligned using the MAFFT v. 7.450 alignment software (Katoh and Standley, 2013) in Geneious version 10.2.6 (Kearse et al., 2012) for preliminary identification of the most conserved ToBRFV genomic regions. Then, a set of ToBRFV-specific primers was designed using the LAMP Designer software (OptiGene Limited, Horsham, United Kingdom). The 5' region of the RNA-dependent RNA polymerase gene (*RdRp*) was chosen as the amplification target. The primer set included two external primers (forward outer primer F3 and backward outer primer B3), two internal primers (forward inner primer FIP and backward inner primer BIP), and two additional loop primers (backward loop primer LB and forward loop primer LF) to augment the number of loops in the LAMP reaction, thus enhancing the reaction speed. The sequences and binding sites of the primers are shown in Table 1 and Figure 1.

**Table 1:** RT-LAMP primers for detection of ToBRFV designed in this study.

Primer name	Length (nt)	Sequence 5'–3'	Nucleotide position	Product size (bp)	Reference sequence
ToBRFV_B3	20	GGACACCGTCAACTAGGA	2,576–2,558	278 (from F3 to B3); 163 (from F2 to B2)	MN815773
ToBRFV_BIP (B1c + B2)	43	CCGTGAGTTCTGAGTCAATGGTT – ATGAGGCTCACCATCTCTTA	2,359–2,382 and 2,457–2,437		
ToBRFV_F3	18	TTGGAGTCTTAGATGTTGCG	2,298–2,318		
ToBRFV_FIP (F1c + F2)	43	CCTTCTCCAACTGTCGCAAGTTA – CACATGCTAGGAAGTACCAC	2,452–2,429 and 2,376–2,396		
ToBRFV_LoopB	22	GCTCAGAACACTGAGGAGATT	2,497–2,518		
ToBRFV_LoopF	21	CTCCATGCTCATCATACTCCAA	2,426–2,404		

For each primer, the nucleotide position related to the reference sequence is reported.

Primers were synthesized by Eurofins Genomics (Ebersberg, Germany), dissolved in qPCR-grade water (Promega, United States) to produce 100 μM solutions, and stored at −20°C. The specificity of each primer was verified by comparing the primer sequences against the NCBI GenBank nucleotide and genome databases using the BLASTn tool. The specificity of the primer set was evaluated using Geneious software by *in silico* analysis of the sequences corresponding to ToBRFV genomic regions defined by the F3/B3 LAMP primers of the ToBRFV reference strain (GenBank Acc. Number MN013188) and the most similar sequences found in GenBank after BLASTn search using the F3/B3 sequence of the reference strain. The selected sequences were imported into Geneious and aligned using the MAFFT software with default settings (Supplementary Figure S1A). In addition, the F3/B3 sequence of the reference strain was also aligned with the sequences of eight tobamoviruses downloaded from GenBank: TMV (Acc. Number. V01408), ToMV (Acc. Number. AF332868), tomato mottle mosaic virus (ToMMV; Acc. Number. KF477193), BPeMV (BPeMV; Acc. Number. DQ355023), TMGMV (TMGMV; Acc. Number. M34077), PMMoV (Acc. Number. M81413), paprica mild mottle virus (PaMMV; Acc. Number. AB089381), and obuda pepper virus (ObPV; Acc. Number. D13438; Supplementary Figure S1B). Further, the F3/B3 amplicon obtained in RT-PCR end-point was sequenced, in order to confirm the identity of the amplicon by BLASTn search.

### RNA Extraction

Total RNA was extracted from fresh leaves, dehydrated leaves, and seeds using the RNeasy plant mini kit (Qiagen, United States), according to the manufacturer’s instructions with minor modification (Foissac et al., 2001). In the case of leaves (both fresh and dehydrated), they were placed in nylon mesh U-shaped bags (Bioreba, Reinach, Switzerland) and homogenized with Homex 6.0 (Promega) in presence of 7 ml (100 mg tissue/1 ml buffer) of GBF (Grinding Buffer Foissac; 4 M Guanidine Isothiocyanate, Sodium acetate 0.2 M, disodium EDTA, 0.025 M, Potassium acetate 1 M and PVP 40 K 2.5%). One milliliter of lysate was withdrawn and 200 microliters of 10% N – Lauril Sarcosine (Sarkosil) were added and mixed. After centrifugation at maximum speed for 2 min (13,000 rpm), 500 μl of the supernatant was recovered and transferred to the QIAshredder sieve columns (Qiagen, United States). Subsequently, the extraction protocol took place in accordance with the kit manual. In the case of the seeds, the same procedure was used but the homogenization step was carried out with the aid of a Mixer Mill MM 200 (Retsch, Torre Boldone, Italy) homogenizer in 10 ml steel jars at a high speed (30 oscillations/s) for 30 s.

To check the quality and integrity of the RNA extracted from leaves and seeds, a one-step real-time RT-PCR reaction using starting concentrations of 10 ng/μl of RNA and a TaqMan dual-labeled probe targeting a highly conserved portion of the plant 18S rRNA was employed, as described by Osman et al. (2017). Results showed a mean Cq value of 12.8 ± 2.6 and 16.8 ± 3.5 for the RNA extracted from the leaves and seeds, respectively. Reactions were performed with a CFX96 (Biorad) thermocycler, following the protocol described by Osman et al. (2017). The RNA quality and quantity were further assessed using a QIAxpert spectrophotometer (Qiagen, Hilden, Germany) and the final concentration was adjusted to ~100 ng/μl.

### RT-LAMP and Visual RT-LAMP Assay

Reverse transcription loop-mediated isothermal amplification reactions were performed and optimized on a CFX96 (Biorad) thermocycler using Isothermal Master Mix (ISO-001) from OptiGene (Horsham, United Kingdom). RNA samples were amplified in 0.2 ml strips of eight tubes for Real-Time PCR (Starlab, Milan, Italy). Each isothermal reaction was performed in duplicate, with a final volume of 20 μl. The optimization of the LAMP protocol took into account the isothermal amplification times (from 20 to 40 min), the mix quantities of Reverse Transcription 5× Master Mix kit (GeneSpin, Italy; from 0.5 to 1.5 μl), and the LAMP primer mixture 10X (2.0–5.0 μl), as well as the individual concentrations of the groups of LAMP primers (at concentrations of 0.2–0.4 μM each of F3 and B3, 0.4–0.8 μM each of LoopF and LoopB, and 0.8–1.2 μM each of FIP and BIP). The cDNA was produced for all RNA targets extracted and was tested in RT-LAMP for all the matrices under investigation, with the aim to have a double operational possibility and relative verification of the results (i.e., both in one-step and two-step RT-LAMP). The cDNA synthesis was performed at 25°C for 5 min and 42°C for 10 min, followed by LAMP amplification cycle. To find the optimal temperature for the LAMP amplification, the reactions were carried out at 58–65°C in a thermal gradient using a CFX96 thermal cycler. In order to evaluate the diagnostic selectivity, the optimized protocol was also applied using a LAMP-dedicated Genie II (OptiGene, Horsham, United Kingdom) thermocycler.

With respect to the visual RT-LAMP protocol, reactions were carried out in duplicate using *Bst* 3.0 DNA polymerase (New England Biolabs, Ipswich, Massachusetts, United States) in a total volume of 20 μl. Optimization of the visual assay was carried out in a similar manner to the real-time assay. In particular, several parameters were taken into account: 1.5–1.8× Isothermal Amplification Buffer, 8–12 U of *Bst* 3.0 DNA polymerase, 6–8 mM MgSO_4_, 1.2–1.6 mM deoxynucleotide triphosphates (dNTPs; GeneSpin, Milan, Italy), 0.15–0.3 μM of HNB, Betaine 0.8–1.2 M (GeneSpin, Milan, Italy), 0.2–0.4 μM of F3 and B3 primers, 0.8–1.2 μM of FIP and BIP primers, 0.4–0.8 μM of LoopF and LoopB primers, and 2 μl test sample [no template control (NTC), or extracted RNA]. Set-up and execution of all LAMP reactions was done on a conventional lab bench using designated pipettes and filter tips, while imaging analysis took place in separate rooms. All experiments were independently replicated at least six times.

Visual RT-LAMP results were observed by the naked eye under natural light and photographed using a conventional smartphone camera. A color change to light blue indicated positive samples, while negative samples remained purple. Moreover, to verify the occurrence of LAMP amplification, RT-LAMP amplicons were analyzed by 1.7% agarose gel electrophoresis in 1x TAE buffer, followed by GelRed (Biotium, Hayward, United States) staining and DNA visualization using a transilluminator. Four microliters of 100 bp DNA ladder (GeneSpin, Milan, Italy) was used as a DNA size marker.

### Specificity and Sensitivity of the LAMP Assays and Comparison With End-Point RT-PCR and RT-qPCR

The specificity of the LAMP primers was validated against four different ToBRFV isolates and by testing cross-reactivity with other tobamoviruses, used as non-target samples (see Section “Virus isolates and plant material”). All samples were tested in duplicate in RT-LAMP and values of threshold, baseline, and reaction efficiency were calculated using the CFX Maestro software (Biorad, United States). Samples with a threshold cycle value (Cq/min) above 30 were ignored.

The diagnostic specificity, for both RT-LAMP and visual RT-LAMP, were calculated using the following formula: D/D + C × 100, where C indicates false positives and D indicates true negatives (EPPO, 2019). The diagnostic sensitivity of both methods was calculated using the formula: % diagnostic sensitivity = A/(A+B) × 100, where A is the obtained positives/expected positives (True positives) and B is the obtained negatives/expected positives (False negatives; EPPO, 2019). The analytical sensitivity (limit of detection, LoD) of both methods was verified using 10-fold RNA extract serial dilutions ranging from 22.5 ng/μl to 0.0225 fg/μl, repeated three times. The same dilutions used to calculate the LoD of the assay were also used in end-point RT-PCR (Alkowni et al., 2019; Ling et al., 2019) and SybrGreen RT-qPCR, in order to compare the degree of sensitivity of the three methods (Tables 2, 3). The SybrGreen RT-qPCR was performed in a total volume of 20 μl, with a concentration of 0.4 μM of F3 and B3 primers and 10 μl of SsoAdvanced Universal SYBR Green Supermix (Biorad, Hercules, United States), using the same CFX96 thermal cycler used for the RT-LAMP.

**Table 2:** Primers used for conventional RT-PCR and RT-qPCR amplification for comparison with the RT-LAMP method proposed in this study.

Primers	Sequence (5'–3')	Length (bp)	Annealing (°C)	Protocol	Reference
ToBRFV-F	AATGTCCATGTTTGTTACGCC	560	58	RT-PCR end point	Alkowni et al., 2019
ToBRFV-R	CGAATGTGATTTAAAACTGTGAAT				
ToBRFV_B3	GGACACCGTCAACTAGGA	278	58	RT-qPCR SybrGreen	This study
ToBRFV_F3	TTGGAGTCTTAGATGTTGCG				
ToBRFV-F (5503)	GAAGTCCCGATGTCTGTAAGG	842	55	RT-PCR end point	Ling et al., 2019
ToBRFV-R (6344)	GTGCCTACGGATGTGTATGA				

**Table 3:** Sensitivity of LAMP used for the ToBRFV detection with different techniques.

Dilutions	RT-LAMP	Visual RT- LAMP	RT-PCR^1^	RT-PCR^2^	RT-qPCR SybrGreen^3^
	Cq means ± SD	Positive (+)/negative (−)	Positive (+)/negative (−)	Positive (+)/negative (−)	Cq means ± SD
22.5 ng/μl	3.80 ± 0.15	+	+	+	5.99 ± 0.34
2.25 ng/μl	5.47 ± 0.11	+	+	+	10.05 ± 0.36
0.225 ng/μl	6.57 ± 0.13	+	+	+	13.81 ± 1.95
22.5 pg/μl	7.71 ± 0.37	+	+	+	18.46 ± 2.20
2.25 pg/μl	8.59 ± 0.07	+	+	+	21.86 ± 1.87
0.225 pg/μl	10.34 ± 0.48	+	+	−	25.18 ± 2.03
22.5 fg/μl	12.22 ± 0.86	+	−	−	28.47 ± 2.14
2.25 fg/μl	16.04 ± 2.75	+	−	−	32.43 ± 1.99
0.225 fg/μl	n/a^4^	−	−	−	n/a
0.0225 fg/μl	n/a	−	−	−	n/a

Mean Cq ± SD = mean of the three threshold cycles of each dilution (Cq) ± standard deviation (SD). Cq values above 30 were considered as negative results.

1 Based on Alkowni et al. (2019).

2 *Based on* Ling et al. (2019).

3 *Based on F3 and B3 primer pair*.

4 n/a = not applicable.

The results obtained with gel electrophoresis, capillary electrophoresis using a Qiaxcel (Qiagen, United States), and for the RT-PCR end-point and melt-curve analyses for RT-qPCR with the F3 and B3 LAMP primers were compared (Table 3).

### Detection of ToBRFV Using RT-LAMP and Visual RT-LAMP Assays on Artificially and Naturally Contaminated Seeds

Tomato and pepper seeds were artificially contaminated with the ToBRFV isolate Sic1/19 by the following method: seeds were disinfected using a diluted solution of sodium hypochlorite, as previously described (Prohens et al., 2008). Then, 100 seeds for each species/variety were contaminated with a leaf extract obtained by macerating 50 mg of infected tomato dehydrated leaves in 1 ml of sterile water in a sterile mortar. Seeds were left in the mortar to macerate for 2 h and then air-dried. Naturally contaminated seeds were obtained from fruits of plants infected by ToBRFV identified in a tomato protected crop from southern Italy (Sicily). Before testing the seeds using the two LAMP assays, seed contamination was verified by checking the presence of viral RNA and virus infectivity, using the total RNA extracted separately from 10 seeds, by end-point RT-PCR, using the method described by Alkowni et al. (2019), and by mechanical inoculation on *Nicotiana tabacum* cv. Xanthi nc. Contaminated tomato and pepper seeds were mixed with clean seeds to generate seed lots with 0% (0/1,000 seeds), 2% (1/50 seeds), 1% (1/100 seeds), 0.1% (1/1,000 seeds), 0.05% (1/2,000 seeds), 0.033% (1/3,000 seeds), 0.025% (1/4,000 seeds), 0.02% (1/5,000 seeds), 0.017% (1/6,000 seeds), 0.014% (1/7,000 seeds), 0.012% (1/8,000 seeds), 0.011% (1/9,000 seeds), and 0.01% (1/10,000 seeds) contamination. Seed lots were ground in a mixer mill and the powder was transferred into sterile plastic bags. Total RNA was extracted and used in the RT-LAMP and visual RT-LAMP assays, as described above. The same seed dilutions used for the two LAMP assays were used for end-point RT-PCR assay (Alkowni et al., 2019; Ling et al., 2019) and SybrGreen RT-qPCR using the F3/B3 primer pair, in order to compare the diagnostic sensitivity in relation to the two LAMP assays. Seeds obtained from tomato and pepper plants found to be infected with ToBRFV were also tested as single seeds or seed lots of 5, 10, or 30 seeds.

## Results

### Optimization of the ToBRFV LAMP Assay

A one-step LAMP assay for the rapid detection of ToBRFV was developed using a set of six primers designed from a highly conserved region of the *RdRp* gene (Table 1). Sequencing F3/B3 amplicons of the four ToBRFV reference isolates revealed 100% nucleotide sequence identity with the corresponding genomic region of the virus isolate used to design the LAMP primers (Acc. Number MN815773).

**Figure 2:**
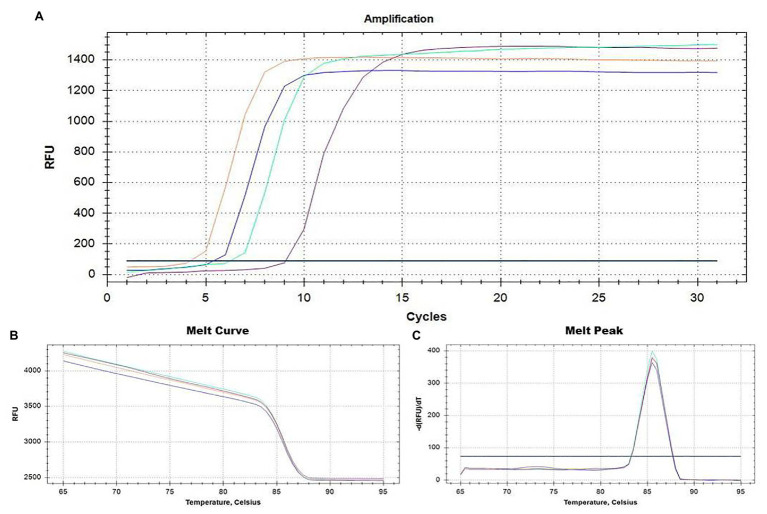
Real-time monitoring of the RT-LAMP result assay for ToBRFV detection based on primer pairs designed on the 5' region of the RNA-dependent RNA polymerase gene (*RdRp*; Table 1). Amplification plots and the two dissociation curves, melting curve and melting peak, are shown in **(A-C)**, respectively. ToBRFV isolates used as positive controls, including Italian isolates Sic1/19 (blue curve) and T1101 (green curve), the Palestinian isolate TBRFV-Ps1 (purple curve), and the German isolate PC-1236 (orange curve). Negative controls (black line) consisted of a no-template control (NTC). Healthy tomato plants and other different tobamoviruses (see section “Virus isolates and plant material”) were also used as negative controls (not shown).

During the optimization of the RT-LAMP assay, significant differences in the Cq values were obtained for all ToBRFV RNA and cDNA with temperatures ranging from 58 to 65°C, resulting in an optimal annealing temperature of 60°C, while negligible differences in Cq values with different concentrations of LAMP primers were observed. The optimal thermal cycle protocol included 5 min of incubation at 25°C, 10 min at 42°C, and 30 min at 60°C, followed a melting curve increasing the temperature from 65 to 95°C with a 1-s interval for every 0.5°C increment. For all the reactions, the melting peak was reached around 86.5 ± 0.5°C. The optimized reaction mix contained 10 μl Isothermal Master Mix, 0.5 μl of Reverse Transcription 5× Master Mix kit, 2.0 μl LAMP primer mixture 10× (final concentrations of 0.2 μM each of F3 and B3, 0.4 μM each of LF and LB, and 0.8 μM each of FIP and BIP), and 2 μl of template RNA diluted 1:10 in sterile water or 2 μl dd-water used as NTC. The results obtained concerning amplification curves, melting curves, and melting peaks by RT-LAMP are shown in Figures 2A–C, respectively.

**Figure 3:**
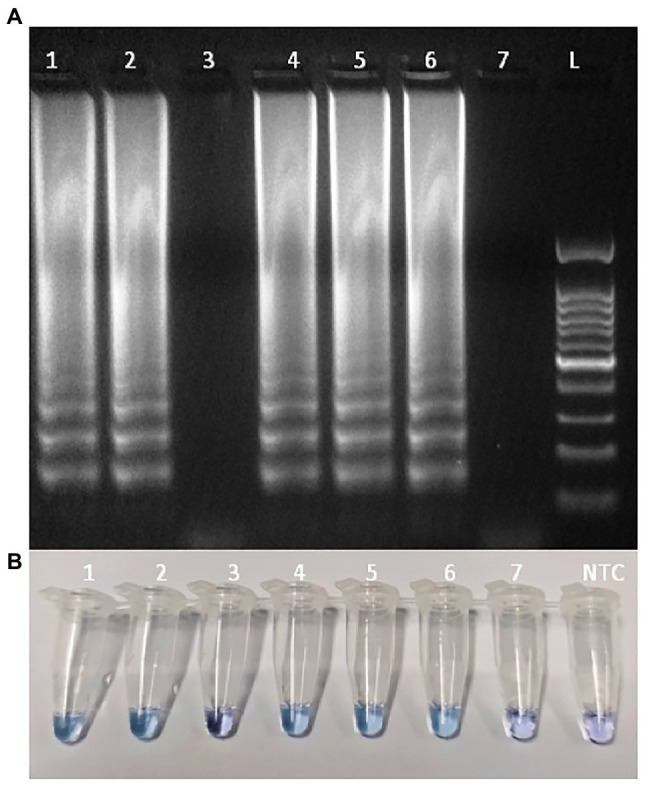
Detection of ToBRFV isolates in tomato leaves samples using loop-mediated isothermal amplification (LAMP) assay: **(A)** Agarose gel electrophoresis analysis of the LAMP products; **(B)** Detecting LAMP products by adding hydroxynapthol blue (HNB) as dye. Lane L: 100 bp ladder (GeneSpin); line 1: isolate Sic1/19; line 2: isolate T1101; line 3: ToMV (isolate PC15705); line 4: isolate TBRFV-Ps1; lines 5 and 6: isolate PC-1236; line 7: tomato healthy leaves; NTC: no template control.

In terms of reaction performance and color change rate, the optimal visual RT-LAMP reaction mixture was as follows: 2.5 μl Isothermal Buffer 10×, 0.6 mM of dNTPs, 2 mM of MgSO4, 0.2 M of Betaine, 0.5 μl of Reverse Transcription 5× Master Mix kit, 2.0 μl LAMP primer mixture 10× (at final concentrations of 0.2 μM each of F3 and B3, 0.4 μM each of LF and LB, and 0.8 μM each of FIP and BIP), 8 U of *Bst* 3.0, 150 μM of hydroxynapthol blue (HNB) dye as a visual indicator, and 2 μl of template RNA diluted 1:10 in sterile water or 2 μl sterile water used as NTC. The optimal thermal cycle consisted of 25°C for 5 min and 42°C for 10 min, followed by one cycle of 60°C for 30 min, and a final cycle of 80°C for 2 min to inactivate the polymerase and terminate the reaction. Using HNB dye as a visual indicator, positive samples produced an intense blue color, while negative samples presented a pale purple coloration (Figure 3).

### Diagnostic Specificity and Sensitivity of the LAMP Assay

The RT-LAMP assay detected all ToBRFV isolates, while none of the non-target tobamoviruses produced any amplification throughout the entire 30 min test (Supplementary Figure S2). A distinct peak on the melting temperature curve (86.5 ± 0.5°C), resulting from the melting curve analysis in RT-LAMP, was found only for ToBRFV isolates, confirming the specificity of the RT-LAMP assay (Figures 2A–C).

For the *Bst* 3.0-based visual RT-LAMP assay using HNB as a colorimetric indicator, all positive reactions showed a color change from purple to blue, while the negative ones remained purple. Thus, the positive and negative results were easily distinguishable by the naked eye. The typical ladder-like pattern of LAMP products was observed only for the ToBRFV isolates in gel electrophoresis, confirming the specificity of the LAMP assay (Figure 3).

Based on the diagnostic specificity tests, performed either using the RT-LAMP or the visual RT-LAMP, the percentage of diagnostic specificity resulted as 100%. The same results were observed for the diagnostic sensitivity, which was equal to 100%.

**Figure 4:**
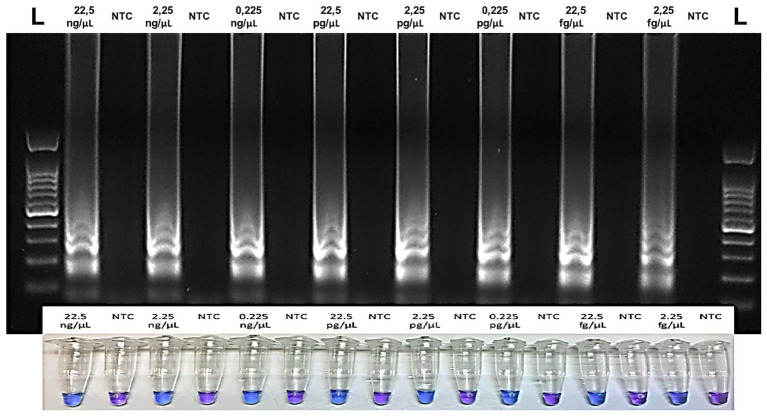
Analytical sensitivity of the Visual RT-LAMP based on the HNB colorimetric assay showing the relative reaction tubes: purple color indicates negative samples, while blue color indicates the positive ones. For each reaction tube, the corresponding agarose gel is shown above. L = 100 bp DNA Ladder (GeneSpin).

**Figure 5:**
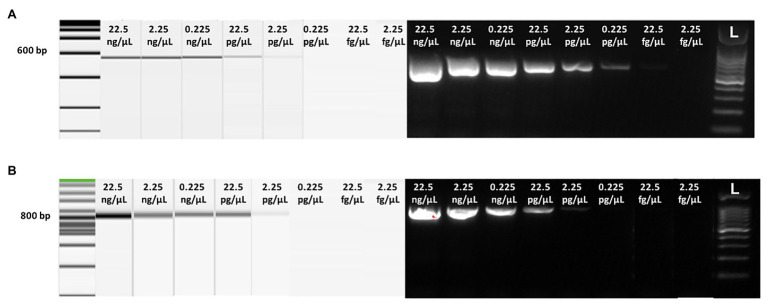
Analytical sensitivity of conventional RT-PCR detected by capillarity and gel electrophoresis using 10-fold serial dilution of purified total RNAs extracted from tomato leaves infected with Sic1/19 isolate of ToBRFV, based on the end-point RT-PCR protocols described by Alkowni et al. (2019; **A**) and Ling et al. (2019; **B**), respectively. On the top of each lane, total RNA concentration is reported. L = 100 bp ladder (GeneSpin).

**Figure 6:**
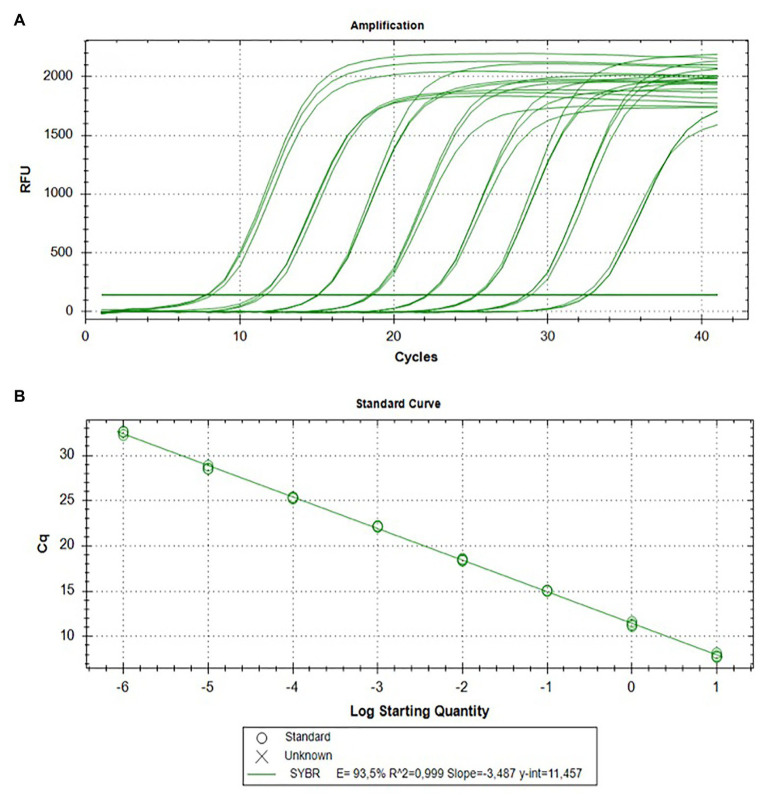
Analytical sensitivity of the qPCR SybrGreen assay using the F3/B3 primer pair for ToBRFV detection. Amplification curves in triplicate **(A)** and the resulting standard curve **(B)** are shown.

To assess the analytic sensitivity (LoD) of RT-LAMP and visual RT-LAMP assay, using the set of primers and to compare the LoD with that of end-point RT-PCR and qPCR SybrGreen, we compared all four methods using 10-fold serial dilutions of total RNA extracted from Sic1/19-infected leaves. In particular, the threshold cycle of each reaction increased along with the dilution degree, and the average values of Cq of three replicates were calculated (Table 3). The Cq values showed a linear relationship with the log value of the RNA concentrations in the 10-fold dilution series (*R^2^* = 0.99). For the 10-fold dilution series, starting from 22.5 ng/μl to a value of 0.0225 fg/μl, we observed that the highest dilution at which LAMP showed positive results for ToBRFV was 2.25 fg/μl (Table 3; Figure 4). The RT-LAMP and visual RT-LAMP assays, were perfectly comparable and 100 times more sensitive than RT-PCR, based on the method described by Alkowni et al. (2019) and 1,000 times more sensitive than RT-PCR, based on the data of Ling et al. (2019), whose LoD results were 0.225 and 2.25 pg/μl, respectively (Figure 5). Finally, the qPCR assay based on SybrGreen and using the F3 and B3 primers showed high performance, with values consistent with those obtained by LAMP assays, thus supporting their specificity and sensitivity (Figure 6).

### Detection of ToBRFV in Tomato and Pepper Seeds

To evaluate the efficiency and feasibility of both LAMP methods in detecting ToBRFV in tomato and pepper seeds, artificially and naturally contaminated seed samples were tested and compared with RT-PCR, based on the results of Alkowni et al. (2019) and Ling et al. (2019), and with qPCR SybrGreen assay based on the F3/B3 primer pair.

**Figure 7:**
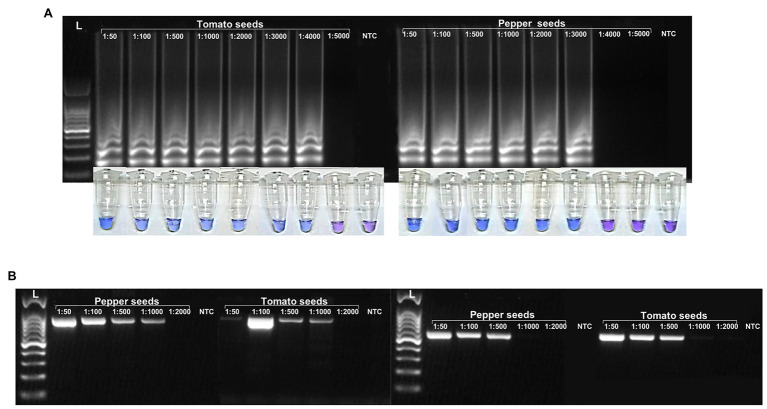
Comparison of ToBRFV sensitivity detection between RT-LAMP **(A)** and RT-PCR, based on the results of Ling et al. (2019; **B**, left) and Alkowni et al. (2019; **B**, right) using 10-fold serial dilutions of purified total RNA extracted in contaminated pepper and tomato seeds.

With artificially contaminated seeds, the RT-LAMP and visual RT-LAMP assay detected ToBRFV up to 0.025% of contamination in tomato seeds (one contaminated seed per 3,000 healthy seeds; Table 4; Figure 7A, right) and 0.033% of contamination in pepper seeds (one contaminated seed per 4,000 healthy seeds; Table 5; Figure 7A, left). Comparable results to the LAMP assays were also observed when the LAMP external primers F3 and B3 were used with the qPCR SybrGreen assay (Tables 4 and 5).

**Table 4:** Detection of ToBRFV in seed-lots of tomato with different techniques.

Seedlots of Tomato	RT-LAMP	Visual RT- LAMP	RT-PCR^1^	RT-PCR^2^	RT-qPCR SybrGreen^3^
	Cq means ± SD	Positive (+)/negative (−)	Positive (+)/negative (−)	Positive (+)/negative (−)	Cq means ± SD
0/1,000	n/a^4^	−	−	−	n/a
1/50	11.10 ± 0.93	+	+	+	26.85 ± 0.2
1/100	12.15 ± 0.67	+	+	+	26.40 ± 0.25
1/500	5.50 ± 0.64	+	+	+	27.12 ± 0.05
1/1,000	8.67 ± 4.55	+	+	+	22.95 ± 0.12
1/2,000	15.13 ± 2.32	+	+	−	23.59 ± 0.08
1/3,000	17.4 ± 0.13	+	−	−	25.10 ± 0.10
1/4,000	15.15 ± 0.70	+	−	−	27.42 ± 0.19
1/5,000	n/a	−	−	−	n/a
1/6,000	n/a	−	−	−	n/a
1/7,000	n/a	−	−	−	n/a
1/8,000	n/a	−	−	−	n/a
1/9,000	n/a	−	−	−	n/a
1/10,000	n/a	−	−	−	n/a

Mean Cq ± SD of seed-lots of tomato = mean of the three threshold cycles of each dilution (Cq) ± standard deviation (SD). Cq values above 30 were considered as negative results.

1 *Based on* *Alkowni et al. (2019)*.

2 *Based on* *Ling et al. (2019)*.

3 *Based on F3 and B3 primer pair*.

4 n/a = not applicable.

**Table 5:** Detection of ToBRFV in seed-lots of pepper with different techniques.

Seedlots of Pepper	RT-LAMP	Visual RT- LAMP	RT-PCR^1^	RT-PCR^2^	RT-qPCR SybrGreen^3^
	Cq means ± SD	Positive (+)/negative (−)	Positive (+)/negative (−)	Positive (+)/negative (−)	Cq means ± SD
0/1,000	n/a^4^	−	−	−	n/a
1/50	8.54 ± 1.70	+	+	+	24.55 ± 0.19
1/100	11.59 ± 1.37	+	+	+	25.80 ± 0.22
1/500	3.51 ± 0.94	+	+	+	26.54 ± 0.16
1/1,000	12.41 ± 0.64	+	+	+	25.87 ± 0.17
1/2,000	13.97 ± 0.42	+	−	−	26.67 ± 0.28
1/3,000	16.24 ± 2.84	+	−	−	27.25 ± 0.16
1/4,000	n/a	−	−	−	n/a
1/5,000	n/a	−	−	−	n/a
1/6,000	n/a	−	−	−	n/a
1/7,000	n/a	−	−	−	n/a
1/8,000	n/a	−	−	−	n/a
1/9,000	n/a	−	−	−	n/a
1/10,000	n/a	−	−	−	n/a

Mean Cq ± SD of seed-lots of pepper = mean of the three threshold cycles of each dilution (Cq) ± standard deviation (SD). Cq values above 30 were considered as negative results.

1 *Based on* *Alkowni et al. (2019)*.

2 *Based on* *Ling et al. (2019)*.

3 *Based on F3 and B3 primer pair*.

4 n/a = not applicable.

The detection limits for conventional RT-PCR were found to be below that obtained with the LAMP and SybrGreen assays: 0.10% of contamination for both pepper and tomato seeds, based on Ling et al. (2019; Figure 7B, left), and 0.10 and 0.05% of contamination of pepper and tomato seeds, respectively, based on Alkowni et al. (2019; Figure 7B, right).

Tomato brown rugose fruit virus was also detected in all seed samples obtained from naturally infected plants of tomato and pepper, both with LAMP methods and by qPCR (data not shown).

The 10-fold serial dilution of total RNA extracted from artificially contaminated seed lots, as inoculated on *N. tabacum* cv. Xanthi nc, led to hypersensitive reaction on the inoculated leaves within 1 week at up to 1:4,000 serial dilution of RNA (data not shown).

## Discussion

Two LAMP assays were developed in this work (RT-LAMP and visual RT-LAMP), with the aim of designing a simple, fast, and also, in the case of visual RT-LAMP assay, cheap diagnostic method, for screening tomato and pepper seeds for the presence of ToBRFV.

These two approaches are both characterized by advantages and disadvantages, and can be combined to adapt the LAMP methodology to various situations (Panno et al., 2020c). For an end-point-based LAMP method – in particular, the visual RT-LAMP developed herein – the most remarkable advantages are its ease to use, and the possibility to use it directly in the field or in laboratories which do not possess specialized personnel and equipment (e.g., thermocyclers). On the other hand, the proposed RT-LAMP method possesses two important advantages: there is no need to check the amplification product at the end of the reaction by the naked eye, and it is easy to read the results though an increase in fluorescence during the amplification reaction. Moreover, by determining the melting temperature of the final reaction product, the result can be further confirmed, thus excluding non-specific products or primer-dimer products. This method showed the same results as conventional qPCR, with the advantage of being more rapid and easy to use. The reaction can be carried out using a typical real-time PCR thermocycler, generating results in <30 min for most samples (Panno et al., 2020c). Considering these aspects, the diagnostic protocols presented in this study were developed based on both RT-LAMP and visual RT-LAMP assays.

Very recently, another LAMP assay for the detection of ToBRFV has been proposed by Sarkes et al. (2020). This assay was applied to plant matrices and gene constructs (gBlocks) to verify their diagnostic specificity. Our work differs for several substantial aspects, including the starting matrices (pepper and tomato seeds), with the relative peculiarities concerning nucleic acid extraction, presence of inhibitors, and diagnostic sensitivity. Furthermore, by developing two LAMP methods in parallel, one in real-time and one visual, it was possible to verify any doubtful or uncertain diagnostic cases through the cross-use of the two techniques. The diagnostic specificity is also confirmed by the use of the melting point (peaks and graphs of the melting curves) of the RT-LAMP, which cannot be verified with the visual RT-LAMP alone.

Finally, in the work presented by Sarkes et al. (2020), specificity was evaluated with respect to only two tobamoviruses, ToMV and TMV, while we used a total of eight different tobamoviruses, including, in addition to ToMV and TMV, other important tomato and pepper viruses, such as ToMMV, BPeMV, TMGMV, PMMoV, PaMMV, and ObPV. It is important to remember that ToMMV, like ToBRFV, has been reported on resistant tomato genotypes (Li et al., 2013; Turina et al., 2016), and therefore, certified and verified LAMP methods are needed to specifically identify only ToBRFV.

Our method based on the two above-mentioned protocols resulted in high specificity, being able to distinguish ToBRFV from the other tobamoviruses with no cross-reactions between the RNA extracts of tomato and pepper and viral RNA. The performance characteristics of the LAMP protocol developed in real-time showed the highest degree of inclusiveness, exclusivity, and diagnostic specificity. These results were also confirmed by the repeatability and reproducibility obtained with different operators (data not shown).

Unlike most of the LAMP protocols that use thermostable polymerases for which a single amplification cycle at high temperatures is adopted, we adopted lower initial temperatures (25°C for 5 min followed by 42°C for 10 min) with the aim of favoring the cDNA synthesis in this phase, probably allowing to reach high sensitivities.

The optimization conditions of the method, based on the use of different reagents and thermocyclers and testing different annealing temperatures and reagent concentrations, did not show any significant differences, in terms of diagnostic results, indicating the robustness of the developed method. Moreover, LAMP assays have shown higher robustness, in terms of pH change, temperature stability, and the use of plant extracts which commonly inhibit PCR reactions (Francois et al., 2011; Kogovsek et al., 2015).

In our experiments, comparison between conventional RT-PCR and the LAMP protocols we developed, demonstrated the higher sensitivity of the LAMP methods. In particular, our LAMP methods were 100 and 1,000 times more sensitive than the RT-PCR methods described by Alkowni et al. (2019) and Ling et al. (2019), respectively (Figures 4, 5). This was demonstrated using both plant leaf (fresh or dehydrated) and seed samples of tomato or pepper (Figure 7). The qPCR SybrGreen method using the F3/B3 LAMP external primers gave similar results to those obtained by RT-LAMP assay, with optimal reaction efficiency parameters (*R^2^*, slope, and E), indirectly supporting the specificity and sensitivity of the RT-LAMP assay (Figure 6).

The LAMP methods developed herein were able to detect as little as 2.25 fg/μl of ToBRFV RNA from host plants, either by RT-LAMP or visual RT-LAMP (Table 3; Figure 4). In addition, LAMP assays performed on pepper and tomato seeds gave good results, in terms of analytic sensitivity, considering the percentage of infected seeds over the healthy ones. The LAMP assays proposed in this study were able to detect ToBRFV to 0.025% of contamination in tomato seeds (Table 4; Figure 7A, right) and 0.033% of contamination in pepper seeds (one contaminated seed per 3,000 and 4,000 healthy seeds, respectively; Table 5; Figure 7A, left), while the detection limits for conventional RT-PCR, were 0.10% of contamination for both pepper and tomato seeds, based on Ling et al. (2019; Figure 7B, left); 0.10 and 0.05% of contamination of pepper and tomato seeds, respectively, based on Alkowni et al. (2019; Figure 7B, right).

In conclusion, the results obtained in the present study show that, using the rapid and versatile LAMP methods developed, accurate and reliable diagnosis of ToBRFV can be performed in leaves and, for the first time, in tomato and pepper seeds. This approach offers a new diagnostic tool in phytosanitary investigations, which can be used to support plant and seed inspection and early diagnosis of ToBRFV infection at official entry points, nurseries, during plant and seed trade, and for the correct implementation of the phytosanitary management of ToBRFV.

## Data Availability Statement

The original contributions presented in the study are included in the article/Supplementary Material, further inquiries can be directed to the corresponding author.

## Author Contributions

DR and GP conceived the work and supervised the experiments. Experiments were performed by AP, DL, GC, LB, and GP. The article was drafted by DR, AP, DL, and GP. All authors contributed to manuscript revision, read, and approved the final manuscript.

### Conflict of Interest

The authors declare that the research was conducted in the absence of any commercial or financial relationships that could be construed as a potential conflict of interest.

## Footnotes

^1^https://www.ncbi.nlm.nih.gov/
