# Supplementary material for: Rapid and Sensitive Detection of Tomato Brown Rugose Fruit Virus in Tomato and Pepper Seeds by Reverse Transcription Loop-Mediated Isothermal Amplification Assays (Real Time and Visual) and Comparison With RT-PCR End-Point and RT-qPCR Methods
Source: Front Microbiol. 2021 Apr 21;12:640932. doi: 10.3389/fmicb.2021.640932 (PMC8096992; doi:10.3389/fmicb.2021.640932)
Supplement: Supplementary file 1 [file Table_1.docx]

**Supplementary Material**

**Figure S1.** Alignment of the sequences corresponding to ToBRFV genomic regions defined by the F3/B3 LAMP primers of the ToBRFV reference strain (GenBank Acc. Number MN013188) and the most similar sequences found in GenBank after BLASTn search **(A)**; sequences of other tobamovirus species present in GenBank **(B).** The positions of the six LAMP primers developed in the study are marked by blue frames.

**(A)**


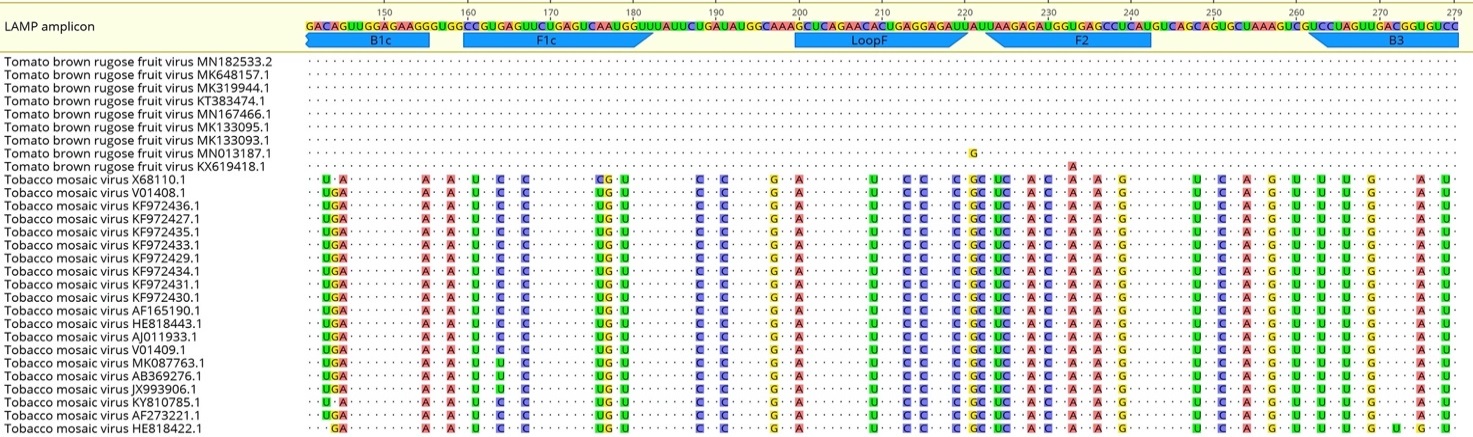

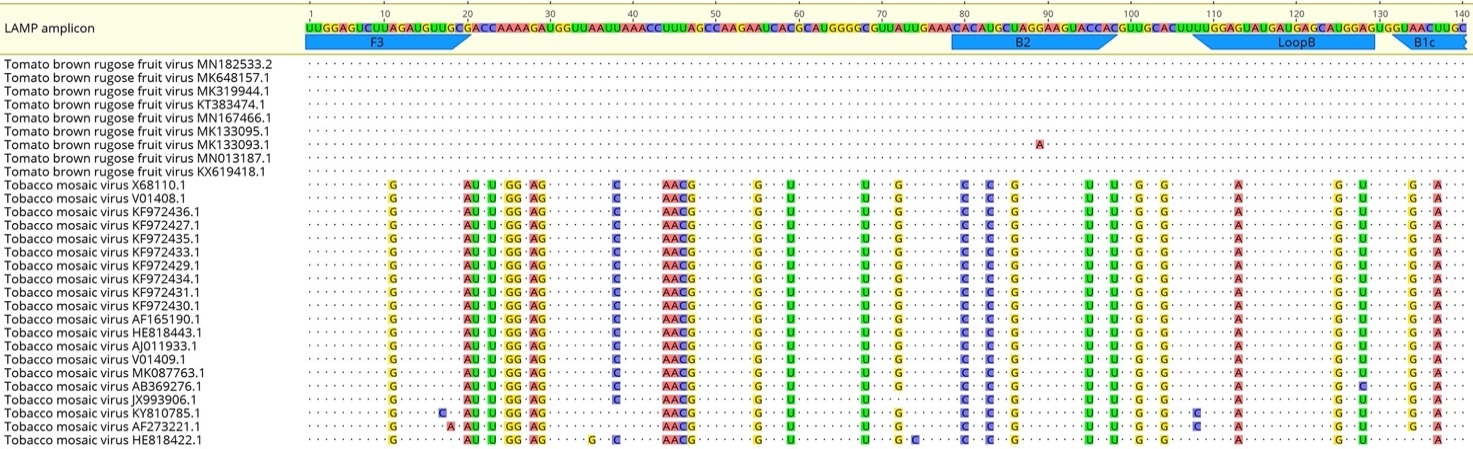


**(B)**

**
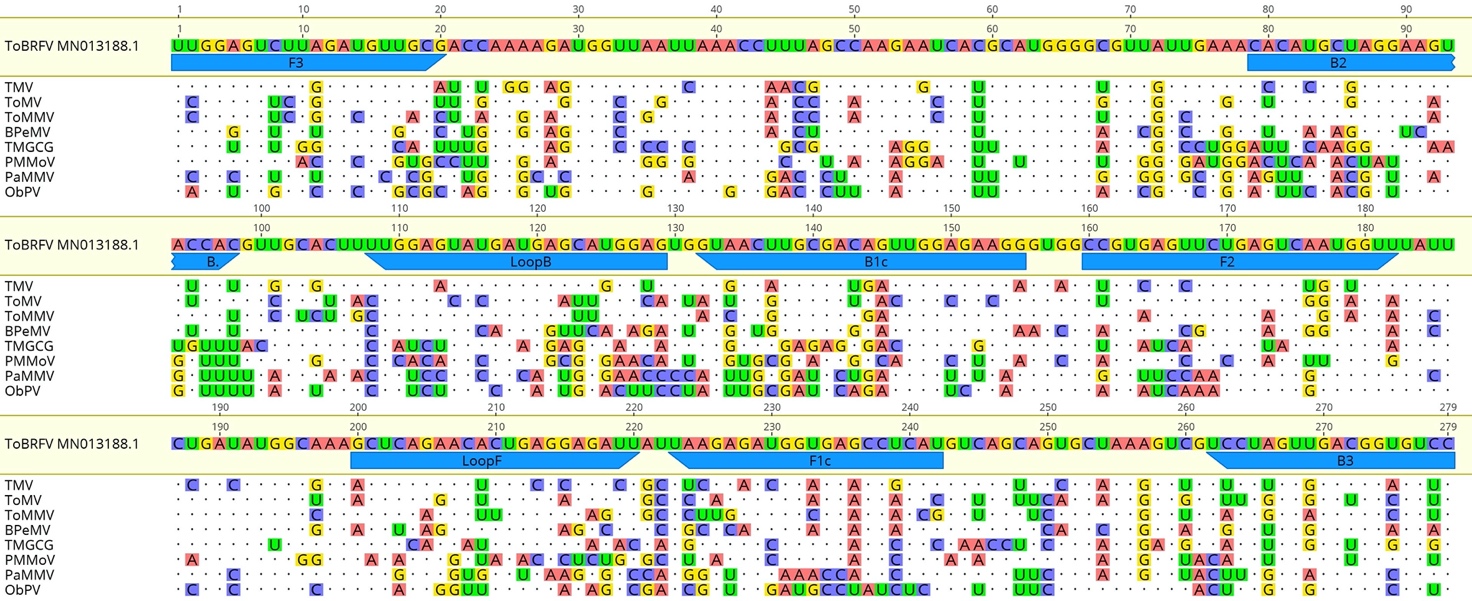
**

**Figure S2.** Amplification plots of the four target ToBRFV isolates (coloured curves with triangles) and eight non-target tobamoviruses (black lines with circles). ToBRFV isolates were: Sic1/19 (red curve), T1101 (blue curve), TBRFV-Ps1 (orange curve) and PC-1236 (green curve).

***
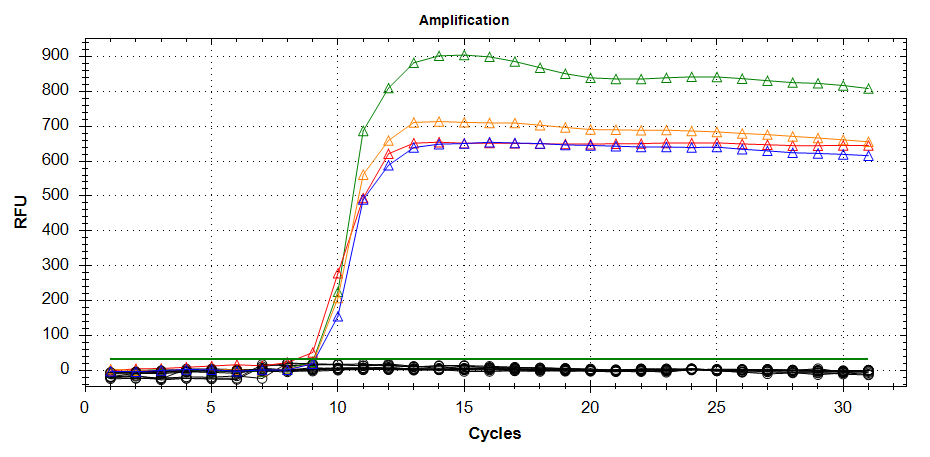
***
